# Agent based modeling of Treg-Teff cross regulation in relapsing-remitting multiple sclerosis

**DOI:** 10.1186/1471-2105-14-S16-S9

**Published:** 2013-10-22

**Authors:** Marzio Pennisi, Abdul-Mateen Rajput, Luca Toldo, Francesco Pappalardo

**Affiliations:** 1 Department of Mathematics and Computer Science, University of Catania, Italy; 2 Bonn-Aachen International Center for Information Technology [B-IT], University of Bonn, Germany; 3 Merck KGaA, Darmstadt, Germany; 4 Department of Drug Sciences, University of Catania, Italy

## Abstract

**Background:**

Multiple sclerosis (MS) is a disease of central nervous system that causes the removal of fatty myelin sheath from axons of the brain and spinal cord. Autoimmunity plays an important role in this pathology outcome and body's own immune system attacks on the myelin sheath causing the damage. The etiology of the disease is partially understood and the response to treatment cannot easily be predicted.

**Results:**

We presented the results obtained using 8 genetically predisposed randomly chosen individuals reproducing both the absence and presence of malfunctions of the Teff-Treg cross-balancing mechanisms at a local level. For simulating the absence of a local malfunction we supposed that both Teff and Treg populations had similar maximum duplication rates. Results presented here suggest that presence of a genetic predisposition is not always a sufficient condition for developing the disease. Other conditions such as a breakdown of the mechanisms that regulate and allow peripheral tolerance should be involved.

**Conclusions:**

The presented model allows to capture the essential dynamics of relapsing-remitting MS despite its simplicity. It gave useful insights that support the hypothesis of a breakdown of Teff-Treg cross balancing mechanisms.

## Background

Multiple sclerosis (MS) is a disease of central nervous system. The disease causes the removal of fatty myelin sheath from axons of the brain and spinal cord resulting reduced communication among nerve cells. Autoimmunity plays an important role in the disease outcome and body's own immune system attacks on the myelin sheath causing the damage. Several genetic factors including HLA-DR15, HLA-A*02 and HLA-DRB1*1501 [1,2] are documented to relate with MS.

MS concordance rate is greater in fraternal twins than for siblings [3,4] showing the involvement of genetic factors. Besides genetic factors, environmental factors are also considered to have a significant role e.g. Epstein-Barr viral infection [5-7] and some dietary factors. Vitamin D and turmeric play a protective role in MS and neurodegeneration.

Turmeric protects brain from neurodegeneration and vitamin D is considered one of the most important factors to prevent MS [8]. Some also considered that there is a significant role of epigenetic factors [9] in the disease occurrence. The symptoms of the disease includes weakness of limbs, vision problems, slurred speech, fatigue, dizziness, difficulty in muscle coordination and losing strength and uncontrolled bodily functions. The disease occurs mostly in the age of 20-40 and women are affected twice compared to men.

The etiology of the disease is partially understood and the response to treatment cannot easily be predicted. Symptoms of MS are apparently unpredictable and may vary from person to person, which represents one of the most disturbing aspects reported by patients. Even in the same patient, symptoms and treatment responses may vary from time to time. Therefore, establishing the prognosis of the disease and predicting the response to therapy at the individual level is a real challenge. Environmental factors (e.g. vitamin D levels, antibodies against Epstein-Barr Virus, smoking), pharmaceutical therapy (immunotherapy) and behavioral factors (stress) may have a significant effect on the clinical course and pathway of the disease.

Currently, more than 400,000 patients suffer from MS in Europe; for the US a number between 250,000 and 400,000 is discussed. MS has a prevalence that ranges between 2 and 150 per 100,000 individuals, depending on the country or specific population [10].

Relapsing remitting multiple sclerosis (RRMS) is the most prevalent type of MS, around 90% of all the patients have RRMS [11], in which disease relapse and remission occurs after a certain time period. Relapse is a term which defines a period of worsening of disease activity, it could be development of new symptoms or reoccurrence of previous symptoms with or without increased severity. Remission is defined as complete or partial recovery of the symptoms following relapse. The occurrences of the relapse vary from mild to severe based on the course and history of the disease. It is also common to have a progressive phase of the disease and a large study showed that around 80% of cases followed by chronic progression within 20 years [12]. Disease progression can be observed by different means including Expanded Disability Status Score (EDSS), Magnetic Resonance Imaging (MRI) lesion and with other physical test including timed 25-Foot Walk, MS Walking Scale-12.

Besides those tests clinical relapses are also considered a mean to see whether patient is improving after given a certain drug or the conditions are getting worse. In some cases relapse was considered as an indication of local inflammatory event [13] but it was shown that the relapse rate decreases as the disease progression [14,15]. Moreover brain compensatory mechanisms can show a recovery in neurologic capabilities (remitting phase) even if unrecoverable neural damage has occurred [16].

T-cell plays a major role in disease progression and it is documented that regulatory T-Cell decreases in the peripheral blood when relapse occurs. On the contrary, number of helper T-cells increases in the spinal fluid. It is also hypothesized that homeostasis of regulatory T cells (Treg) and effectors T cells (Teff) play a crucial part in preventing autoimmunity [17-19]. In particular, lack of functionality or deficiency of Treg may entitle negative effects in the peripheral tolerance mechanisms that are believed to control activation and proliferation of Teff [20].

MS is a complex disease involving many biological scales, from molecular scales to organs, and environmental factors. In this scenario mathematical/computational models are requested both in knowledge discovery to perform in silico experiments and suggest preclinical experiments and in clinical scenarios to help Medical Doctors to envisage the correct therapy for their patients.

To build a mathematical/computational model one can use a variety of different techniques [21]. Among these, Agent Based Modeling (ABM) look to be one of the most appropriate to describe complex systems in a flexible way.

In the present paper we propose the first attempt to model MS using ABM. To our best knowledge only Read et al. presented in [22] an ABM for modeling Experimental Autoimmune Encephalomyelitis (EAE), a mouse proxy for MS.

Our model is aimed to describe RRMS taking into account the cross-regulation between the two cells populations, coupled with an external agent (such as the Epstein-Barr Virus (EBV)) supposed as the cause of chronic inflammation, in order to see whether such system is able to show stable oscillatory behaviors in healthy patients, and presence of unrecoverable neural damage in patients with a malfunction in the cross-regulation mechanisms between Teff and Treg.

The present model is rather simple as include only three classes of entities. However, as mentioned before, ABM models are very flexible and the present model can be expanded to include a more detailed description of the immune system entities and functions or environmental factors (i.e., Vitamin D).

## Materials and methods

### Biological hypotheses and conceptual model

To model and simulate RRMS we made some hypotheses based on most recent experimental evidence. The first hypothesis is about the presence of a mechanism of competition and cross regulation between Treg and Teff cells. In particular we supposed that Teff are down regulated by Treg cells and Treg cells are upregulated by Teff cells. Many known mechanisms induce the inhibition of Teff by Treg, for example cell-to-cell contact inhibition [23] and secretion of immunosuppressive cytokines [24]. On the other hand, it has been observed that Treg benefit of a positive feedback which promotes proliferation [25] coming from signals of Teff cells [19].

This cross regulation between the two populations reminds to the famous predator-prey (Lotka-Volterra) equations. In this dynamical system, the population of prey is represented by activated Teff cells that use available food resources (represented by myelin and cerebral tissues) to grow. On the other hand the population of predators (represented by activated Treg) try to catch and suppress them.

Another biological hypothesis that has been carried into account is represented by the fact that an environmental agent such as a virus causes the inflammation. In particular the EBV satisfies all requirements as candidate trigger: it is ubiquitous in nature, establishes a lifelong dormant infection with activation that causes continuous virus production, and modulates the human immune system [26]. Many potential mechanisms have been identified to be responsible for the association of EBV infection with MS [26]. Among these we took into account the possibility that EBV-specific T cells could cross-react with autoantigens expressed in the CNS (such as the myelin basic protein or MBP) and attack the myelin sheath of axons.

We further supposed that there exists a correlation between the presence of a relapse and neural damage. Some recent studies showed in fact that some biomarkers of axonal damage (NFL) and demyelination (MBP) were increased in all RR MS patients [27].

Even if the appearing of a disability cannot be always directly correlated with an inflammation in the central nervous system, many studies using magnetic resonance spectroscopy suggest that axonal loss begins at the onset of the disease and show the presence of brain atrophy in the earliest stages of MS. Moreover it has been shown that brain atrophy increases during the relapsing-remitting disease stage without concurrent disability progression, suggesting that compensatory mechanisms may allow the recovery of neurologic capabilities, even if brain unrecoverable tissue loss during the early stages of the disease occurred [16]. We should note that the observation of (two or more) lesions in different parts of the CNS using magnetic resonance imaging techniques represents nowadays one of the necessary conditions associated with the diagnosis of MS. To this end we supposed to associate the appearing of a relapse with the presence of new unrecoverable neural damage.

Another hypothesis that has been taken into account is given by the fact that, in susceptible individuals, self-reactive T cells are able to pass the thymus selection and are thus present in the blood periphery [11]. Many studies have confirmed the presence of some genetic predisposition by individuating multiple specific genes that entitle higher risks of developing MS. Among these we recall the HLA-DR and -DQ genes and the HLA-DR15 haplotype in Caucasians [28]. The presence of some breakdown in the peripheral immune tolerance mechanisms that allows the activation of Teff [20,29-32] represents another condition that has been taken into consideration. This scenario has been observed both in mice [33,34] and in humans [35,36]. Furthermore it has been observed in MS and type 1 diabetic patients the presence of a dysfunction of the Treg function and an imbalance in Teff-Treg cross-regulation mechanisms [37,38]. This has been modeled by supposing that Treg had lower duplication rates than Teff in hill patients.

### Agent based modeling approaches

Agent-based modeling approaches simulate the behavior of autonomous entities (this could be buyers in economic models, in our case the autonomous entities are cells). The models simulate the simultaneous operations and interactions of multiple agents (in our case cells and molecules), in an attempt to re-create and predict the appearance of complex phenomena (in our case: cellular interactions in neuroinflammation and neurodegeneration). The process is one of emergence from the lower (micro: cell) level of systems to a higher (macro: organ) level.

Agent dynamics can be described as a function of time, position and internal states (i.e. age) of every agent. Interaction (i.e. cooperation or competition) with nearby agents modifies the dynamics of interacting agents. Emerging complex behavior is obtained by taking into account all the microscopic stochastic interactions of all agents which cooperate and/or compete to achieve a global solution.

ABMs naturally handle entity heterogeneity and physical space, and suffer less from the issue of directly designed dynamics. Moreover its easy to describe complex behaviors just because specifying agent rules is intuitively straightforward. On the other and, ABM lack of a solid mathematical basis that could be used to easily study asymptotic behaviors and provide further mathematical analysis and they need massive computational resources in order to allow (near-to) natural scale simulations. High effective parallel algorithms or platforms to support such approaches represent one of the next future major challenges.

ABMs have been successfully used for simulating many pathologies such as HIV virus [39-41], mammary carcinoma and lung metastases [42,43], the cell-based immune response to cancer cell antigen presentation [44] and atherosclerosis [45]. We considered the agent-based modeling technique the ideal approach to model cellular interactions as they occur in neuro-inflammatory and neuro-degenerative processes such as MS.

An easy way to implement an ABM is to use of an agent based oriented programming language. One of these languages is represented by NetLogo. NetLogo is a programming language and integrated modeling suite totally oriented and devoted to ABMs. It was developed in Java by Uri Wilensky in 1999 and it has been continuously updated ever since. It features an extensive documentation and multiple tutorials and a worldwide community that furnishes great support. It is free and open-source and cross-platform. NetLogo represents a good choice to simulate multi-agents, networks and complex dynamical systems [46]. Another choice for developing the ABM model was represented by the use of a general purpose computer language, for example ANSI C. We used such an approach several times [47-52]. However, NetLogo allowed a faster developing of the model as, at this stage, we did not manage a large quantity of details, entities and interactions. Moreover models developed in NetLogo can be easily shared as Java applets i.e., they are able to be run in almost (if not all) computer platforms. Finally NetLogo models take advantage of the graphical interface that easily allows the plot of various entities dynamics. Of course the implementation of a more detailed model would require the flexibility and the speed given by the use of a pure programming language. For huge simulations, parallel computing is highly advisable and the use of a programming language as C is imperative.

### The model

#### Preliminary considerations

In order to simulate RRMS we used Netlogo agent based modeling framework. To this end we built a model that uses a grid of 51x51 cells (namely patches) to simulate a small portion of white matter. Every patch is colored in light gray and is initialized at the beginning of the simulation with non-zero quantity *init_mye *of myelin that covers the axons in healthy patients. In order to simulate recoverable and unrecoverable damage, we supposed that every patch that is attacked by Teff loses a given value of myelin *ate_mye*, in this case the patch is colored in dark gray to simulate the presence of damage. If the patch still contains a quantity of myelin that is greater than zero, the damage can be recovered (recoverable damage) at every time-step with a given rate *rec_mye *up to the initial quantity *init_mye*; otherwise (i.e. the remaining quantity of myelin is zero) the patch is colored in black and the damage cannot be recovered anymore (unrecoverable damage). This mechanism is supported by several facts in reality. Resident oligodendrocytes (if still present) are activated and are able to restore the lost percentage of myelin [11]. We used a time-step of Δ(*t*) = 2.4 hours. We chose such a time-step because it allowed to keep, from a temporal point of view, a good degree of granularity that enabled to simulate singular relapses, yet allowing to reproduce, using reasonable computational resources, the progress of the disease in a time-window of five years (18250 time steps).

The model supposes the presence of three kinds of agents (called turtles in Netlogo): auto reactive effector T cells (Teff), regulatory T cells (Treg), and EBV (Viruses). All agents are introduced with a life counter randomly set to a value between [1; 2*hlife], where *hlife *(Δ(*t*)^*-*1^) represents the mean half life of involved agents. The life counter is usually decremented by 1 at every time-step for all agents. All agents can move and interact during the simulation at every time-step. Movement and interaction rules are described in the next sections. We used a Von Neumann neighborhood [53].

Introduction of all agents inside the simulation is done using stochastic pulse trains instead of Gaussian white noise. As suggested in [54], the introduction of agents using stochastic impulses is advisable in order to gain more realistic and general understanding of the effect of environmental fluctuations leading to extinction of the species. Gaussian white noise assumes the presence of a continuous perturbation, and this is not in line with thymic selection where the selection newborn resting T cells with a given specificity can be seen a sequence of discrete stochastic events. As in the paper by Vélez de Mendizábal et al. [13], both the introduction of resting Teff and Treg is done using trains of 100 randomly distributed impulses per year. This reflected the fact that the generation of a given self-reacting T-cells can be seen as a consequence of unrelated stochastic events [55]. Also viruses are introduced with the same technique.

We would note here that we actually modeled a single organ representing a piece of brain tissue, instead of modeling all the organs and spatial mechanisms (i.e. lymphnodes, central nervous system, blood brain barrier so on) that are involved in the development of MS. This is clearly a simplification of the reality but it did not represent a problem since in the present model activated Teff and Treg are the sole agents that actively interact in and with the brain tissues. The other agents (resting Teff, resting Treg, and viruses) do not interact with the underlying patches (which contain the myelin) and thus it is possible to imagine that all the interactions involving such agents occur elsewhere.

#### Logic implemented in the prototype

**Figure 1:**
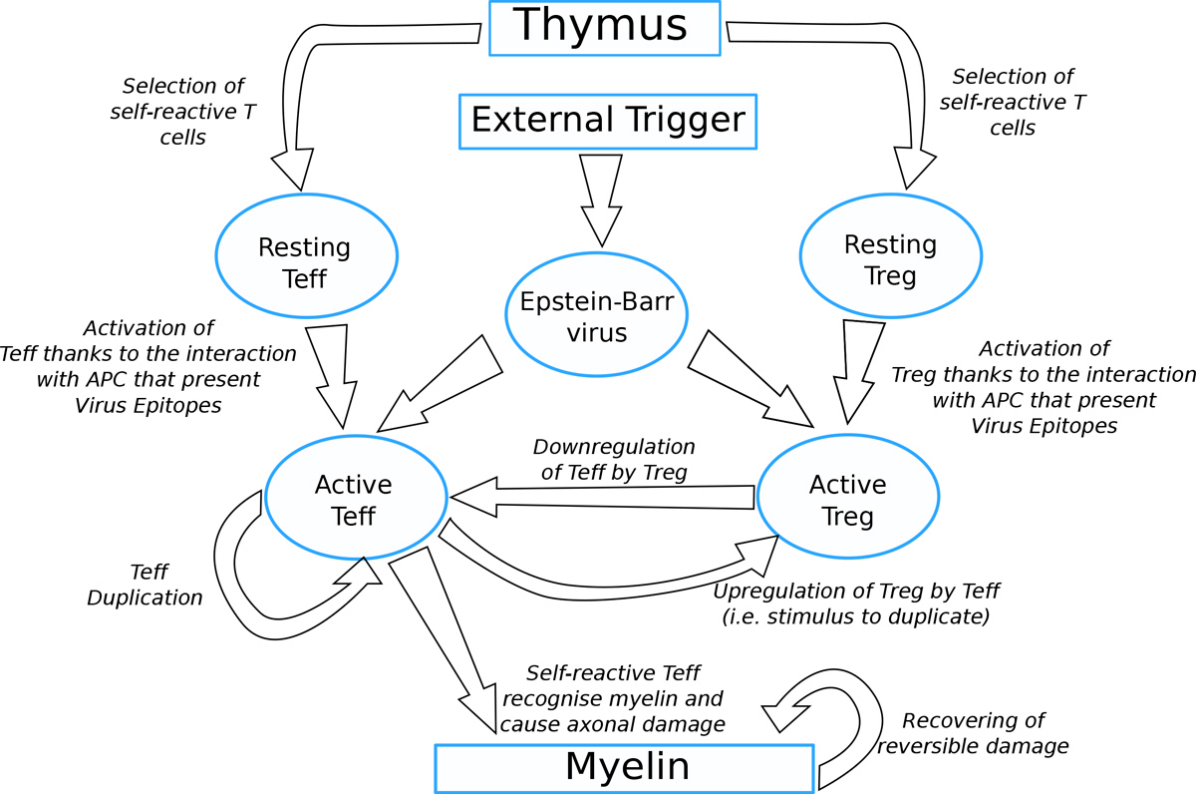
**Conceptual model**. Figure shows the logic implemented into the model, including the genetic predisposition in developing the pathology (i.e. thyms selection of self-reactive clones), the presence of an external trigger (i.e. EBV), the supposed malfunction of the Treg-Teff mechanism, and the effects caused by the malfunction (i.e. neural damage due to myelin loss). Self-reactive resting Teff and Treg cells are introduced by Thymus selection. An external factor such as EBV latent infection can cause activation of both Teff and Treg cells. Activated Teff cells can then attack myelin causing damage in the brain and duplicate, activated Treg will downregulate Teff receiving a positive feedback from that. reversible damage can be recovered.

Due to a genetic predisposition, self-reactive resting Teff and Treg cells are introduced into the system randomly using stochastic pulse trains. The presence of an external factor such as the presence of EBV latent infection can, through mimicry, cause activation of both Teff and Treg cells. Activated Teff cells can then attack myelin in the brain and duplicate, activated Treg will try to catch active Teff and stop their activities, thus they receive a positive feedback and will duplicate. In Figure 1 we show the conceptual model that implements this logic.

#### States and Transitions

Both Teff and Treg cells have two internal states: (a) resting and (b) activated. All cells are introduced into the simulation in resting state. Resting cells cannot duplicate, activated cells can. We suppose for now that viruses have no internal states and are used to trigger the activation of auto reactive T cells through mimicry [56].

All local state transitions entitle global evolving of the system. These state transitions can be described locally as events that occur and modify the state of one or more agents. They mainly happen two reasons: natural state-transitions (i.e. death) and interactions among agents. Both the transitions are presented next, as follows:

• **Death: **At any time step the life counter of any entity is decremented by 1. If the life counter of an agent reaches 0 then the agent is removed from the simulation.

• **Movement: **At any time step all turtles can move in a patch in their neighbourhood chosen at random.

• **Introduction of new agents: **As already discussed, at every time-step 3 bernoulli events (for Teff, Treg, and viruses) with a probability *p *= 100*/*365 are simulated in order to decide whether introduce new turtles or not (stochastic pulse trains). If success occurs the new turtles are introduced into the simulation. Treg and Teff are introduced in resting state.

• **Myelin attack by active Teff: **If active Teff is in a patch with a nonzero quantity of myelin then the Teff will attack the myelin causing axonal damage and then the quantity of myelin will be lowered by *ate mye*; the Teff will be eligible to duplicate. If the quantity of myelin reaches 0 then the Teff will be not able to duplicate.

• **Duplication of active Teff: **If a Teff successfully causes axonal damage, it will be stimulated to duplicate. Duplication is modeled as a stochastic bernoulli process. In other words the Teff will have a probability to duplicate *p_e _*and to do not duplicate equal to 1 *- p_e_*. The probability *p_e _*is calculated for every duplication process according to the following law:

$$p_{e}=eff-dup\cdot \frac{myelin^{2}}{init\text{\_}mye^{2}}\cdot \frac{mean\text{\_}Tregs}{Treg\text{\_}here+mean\text{\_}Tregs}$$

where *myelin *indicates the quantity of myelin in the current patch, *eff_dup *is a duplication constant representing the maximum duplication rate of Teff, *mean_Tregs *is a given threshold and *Treg_here *is the number of Tregs in a given radius *Treg_radius*. The term $\frac{myelin^{2}}{init\text{\_}mye^{2}}$ gives higher probabilities to duplicate if the patch has higher quantities of myelin, whereas the term $\frac{mean\text{\_}Tregs}{Treg\text{\_}here+mean\text{\_}Tregs}$ is used to model the down-regulation of Teff duplication rates by Treg actions. As discussed before, Tregs can inhibit Teff duplication through cytokines signaling. We did not model explicitly these cytokines, but we took into account the possibility an active Treg can release them. So, there is a high number of Tregs in a given radius, then the possibility of having such cytokine inhibitors will be higher and so this will lower the probability of duplicating of Teff.

At this point If the number of agents is below a given threshold *patch_density *then the turtle will duplicate; the turtle will move to a patch in the neighborhood and a new duplication probability *p_e _*will be calculated otherwise. If duplication occurs the duplicating Teff will have its life counter reduced by half. The newborn Teff will be already active and will be positioned in a patch in the neighborhood chosen at random.

• **Active Treg - Active Teff interaction: **If an active Treg finds an active Teff in a neighborhood of radius *treg_radius *the Treg will move towards Teff and will suppress it. Then the Treg will be eligible to duplicate.

• **Duplication of active Treg: **If a Treg successfully suppresses a Teff, it will be stimulated to duplicate. Even Treg duplication is modeled as a stochastic bernoulli process. Treg will have a probability to duplicate *p_t _*and to do not duplicate equal to 1 *- p_t_*. where *p_t _*is a duplication constant representing the maximum duplication rate of Treg. If the number of agents is below a given threshold *patch_density *then Treg will duplicate; the Treg will move to a patch in the neighborhood and a new duplication probability *p_t _*will be calculated otherwise. If duplication occurs the duplicating Treg will have its life counter reduced by half. The newborn Treg will be already active and will be positioned in a patch in the neighborhood chosen at random.

• **Resting Teff - virus interaction: **If a resting Teff encounters a virus in a neighborhood of radius *virus_radius *the Teff switches its internal state to active. The virus will disappear from the simulation. This interaction is used to mimic the activation of auto reactive effector T cells by antigen presenting cells (APC) that present EBV epitopes and it is due antigenic mimicry mechanisms described earlier. We did not actually model APC since at the present time their presence in the model is not strictly fundamental. Successive versions of the model will cover this.

• **Resting Treg - virus interaction: **If a resting Treg encounters in virus in the neighborhood of radius *virus radius *the Teff switches its internal state to active. The virus will disappear from the simulation. This interaction is used to mimic the activation of auto reactive regulatory T cells. Even in this case the same considerations made for Resting Teff - virus interaction hold.

#### Simulation of different individuals

In order to simulate different patients we initialized the Netlogo pseudo-random number generator with different seeds for every simulation. When the pseudo-random number generator is initialized with a given seed it will generate a (replicable) chain of random numbers that are used to decide the occurrence (or not) of stochastic events. So every seed will entitle a completely different sequence of events for each simulation and two simulations that are started with different seeds usually enable totally different observable behaviors and results.

## Results and discussion

As we discussed in section Materials and methods, we supposed that the appearing of a relapse and the presence of new unrecoverable neural damage are correlated [16].

In this paper we present the results obtained using 8 genetically predisposed randomly chosen individuals. We reproduced both the absence and presence of malfunctions of the Teff-Treg cross-balancing mechanisms at a local level. For simulating the absence of a local malfunction we supposed that both Teff and Treg populations had similar maximum duplication rates. In other words we set the maximum duplication rate of Teff *eff_dup *and the duplication rate of Treg *p_t _*to the same value, so both the cells populations have the same maximum duplication rates. We further supposed that the breakdown of the cross regulation mechanism is due to a lower duplication rate *p_t _*of Treg. In Table 1 we reassumed the most important parameters used for the simulations.

**Table 1:** Principal model parameters

Parameter	Value (estimate)	Meaning
*treg_radius*	3	max visibility radius of Treg

*eff_dup*	0.1 (range 0.025 - 0.2) Δ(*t*)^*-*1^[13]	max. duplication rate of Teff

*init_mye*	100	initial quantity of myelin per patch

*eat_mye*	5	quantity of myelin destroyed by Teff

*p_t_*	0.1 in healthy patients 0.025 in hill patients (range 0.025 - 0.2) Δ(*t*)^*-*1^[13]	max. duplication rate of Treg

*patch_density*	3	max. no of entities per patch allowed to have duplication

*Teff_life*	60 (range 50-70) [58] Δ(*t*)	Teff mean half-life

*Treg_life*	60 (range 50-70) [58] Δ(*t*)	Treg mean half-life

We also tested the model by simulating 100 randomly chosen virtual patients (data not shown) in both the hill and the healthy scenarios by setting [*eff_dup *= 0.1; *p_t _*= 0.025] and [*eff_dup *= 0.1; *p_t _*= 0.1], respectively. We took, as outcome of the experiments, the total damage at the end of the simulations for both the scenarios. Median values of the final total damage were 77268 for the hill sample and 5357 for the healty sample. Non parametric Kolmogorov-Smirnov two samples goodness-of-fit test gave as result a maximum difference between the cumulative distributions *D *= 0.7500 with a corresponding *p*-value of: 0.000, thus suggesting that the two samples are unlikely to be drawn from the same distribution (i.e., they are statistically different).

**Figure 2:**
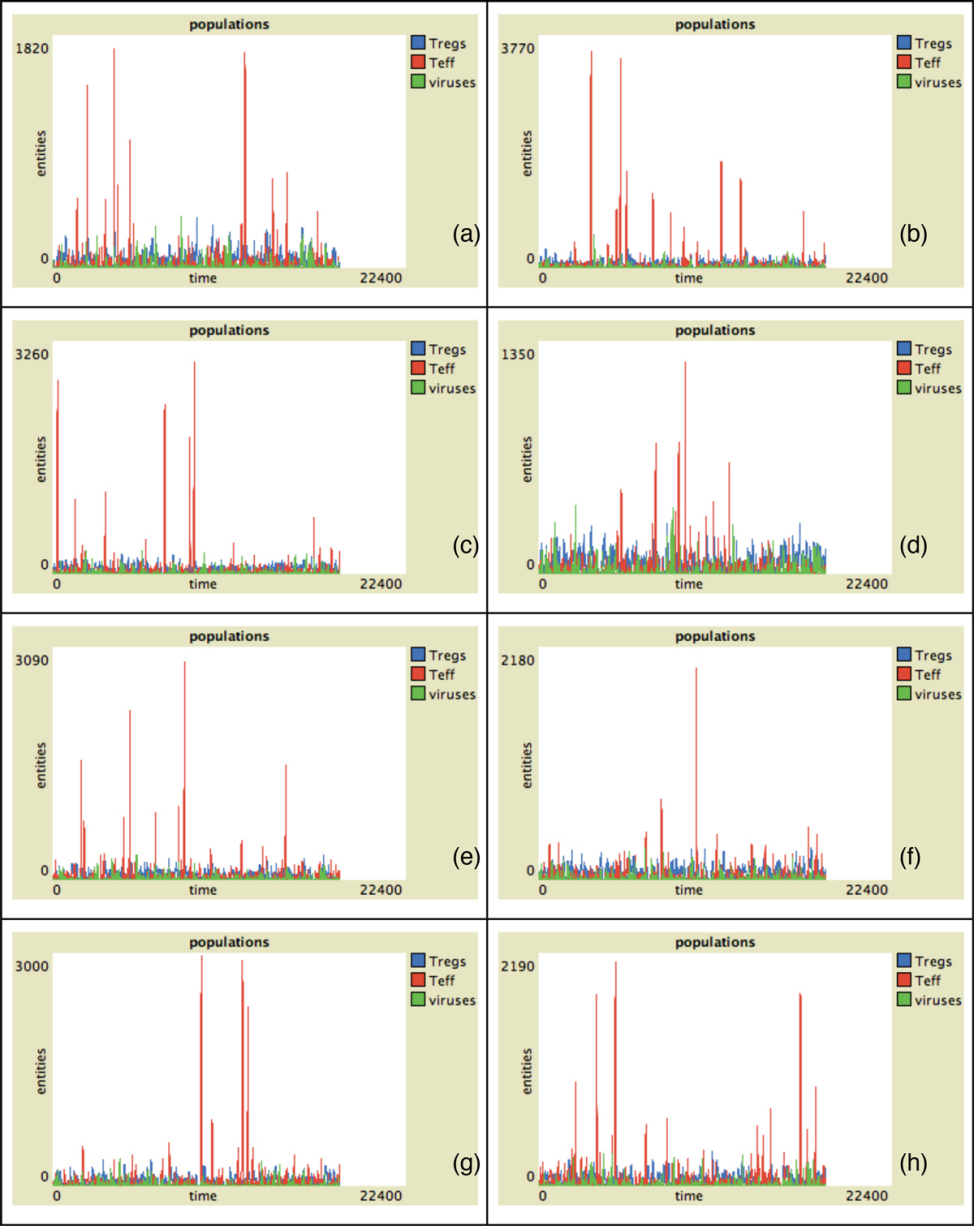
**Entity behaviors vs time in healthy patients**. Simulation of eight randomly-selected healthy virtual patients. We suppose that both Treg and Teff have similar duplication rates. Simulation time is 5 years (18250 time-steps). Red lines represent activated Teff behaviors, blue lines represent activated Treg behaviors and green lines viruses behaviors. In this case the number of Teff peaks is relatively small due to the action of regulatory mechanisms. This would entitle lower probabilities of having unrecoverable damage.

In Figure 2 the behavior of Teff, Treg and viruses vs time for all the presented individuals in absence of malfunctions of the Teff-Treg cross-balancing mechanisms is showed. The number of Teff (in red) has some spikes at different time intervals in all plots. This indicates that in some cases, due to the stochasticity in the introduction of newborn cells, self-reactive Teff may initially escape from Treg control (blue lines) and can be activated due to mimicry, duplicate and try to attack myelin. However activated Treg are able to counterbalance Teff actions maintaining immune homeostasis. This is visible in Figure 4 where we show the levels of damage (recoverable, unrecoverable and total) for all the simulations. In this figure it is possible to see the presence of some spikes in the recoverable damage plots (blue lines) that obviously correspond to the spikes seen in Figure 2. However such damage is usually recovered and at the end of the simulation almost no unrecoverable damage (red lines) arises. Figure 6 presents the spatial plots at the end of every simulation (after 5 years) in healthy patients. The plots confirm the observations that came from Figure 2, as almost no black patches (which indicate the presence of some scarring or lesions) are present.

**Figure 3:**
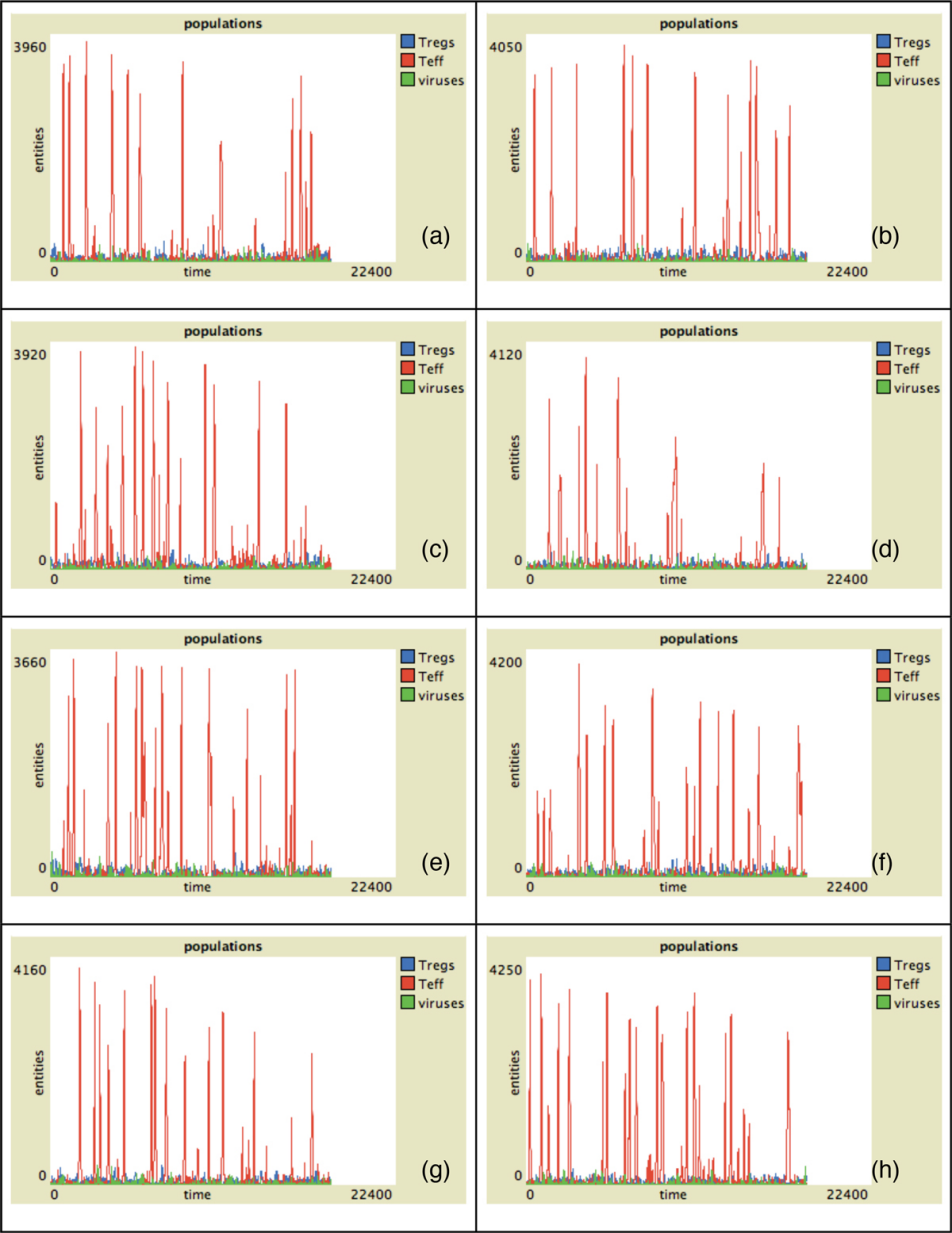
**Entity behaviors vs time in hill patients**. Simulation of eight randomly-selected hill virtual patients. We suppose that the breakdown of the cross regulation mechanism is due to a lower duplication rate *p_t _*of Treg. Simulation time is 5 years (18250 time-steps). Red lines represent activated Teff behaviors, blue lines represent activated Treg behaviors and green lines viruses behaviors. In this case the number of Teff peaks is higher. Moreover each peak reaches higher values in respect to healthy patients, thus indicating that higher numbers of self-reactive Teff may entitle higher probabilities of having unrecoverable damage.

In Figure 3 we show the behavior of Teff, Treg and viruses vs time for all the individuals in the case of malfunctions of the Teff-Treg cross-balancing mechanisms. Similarly to what has been observed in Figure 2, all plots show some spikes in the Teff behaviors (red lines). However in this case the spikes are more numerous and reach higher values thus suggesting that, due the malfunction in the regulatory mechanisms, Teff can be easily activated and cause brain damage. In this case Treg are not always able to contrast Teff actions and maintain homeostasis, and this is visible in Figure 5 where the levels of damage are shown for all the simulations. In this case the spikes in the reversible damage plots (blue lines) are higher and bigger in number. It is also possible to observe the appearing of unrecoverable damage that indicates the appearing MS plaques and to see how the sum of both (total damage, black plots) mimics the typical relapsing-remmiting dynamics observed in MS. Presence of scarring can be also seen in Figure 7 where the spatial plots for all individuals are presented at the end of 5 years. In all plots it is possible to see many black areas that indicate unrecoverable damage and thus the presence of lesions and scarring that may be correlated with relapses and appearing of inability.

**Figure 4:**
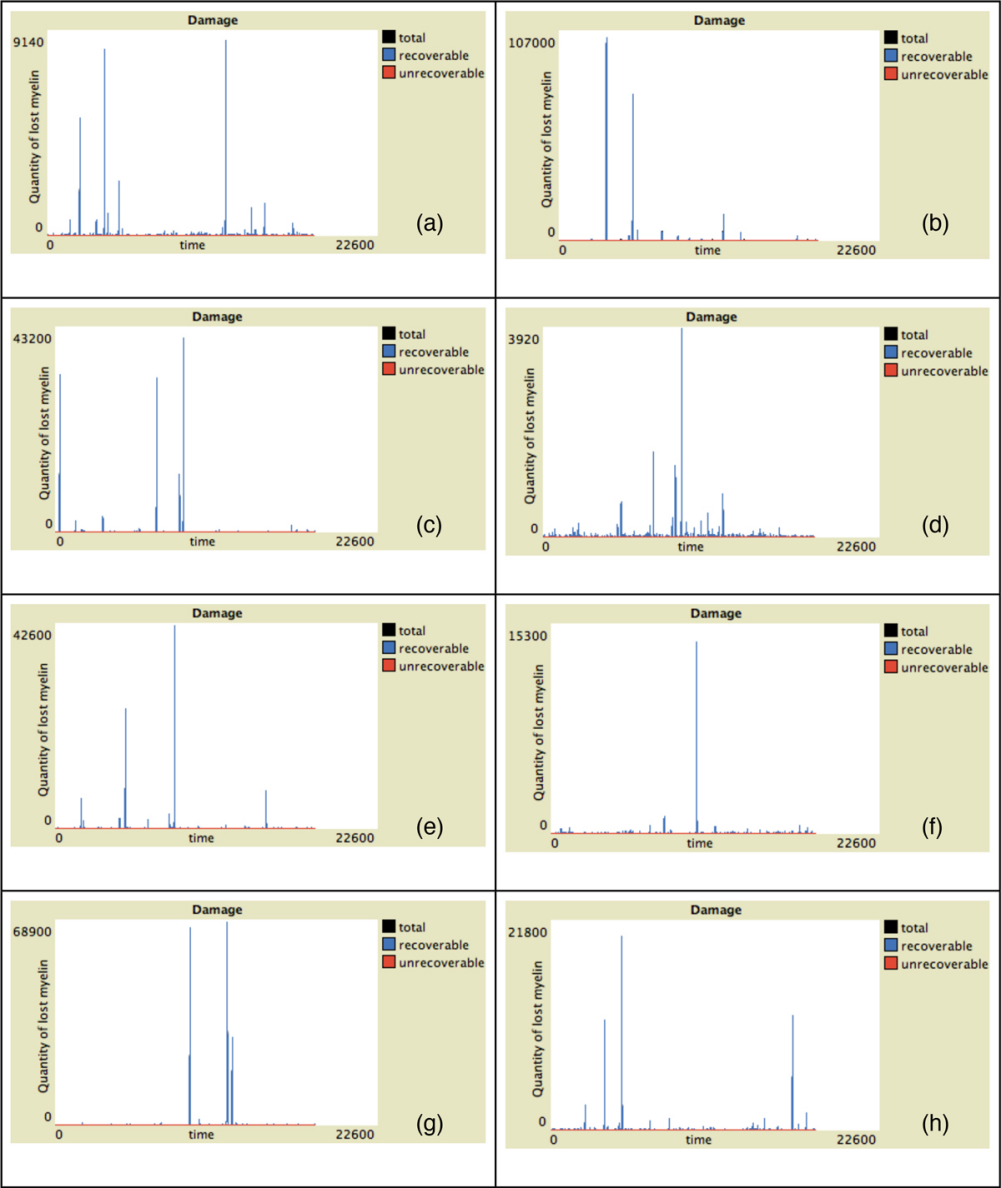
**Damage progression vs time in healthy patients**. Simulation of eight randomly-selected healthy virtual patients. Simulation time is 5 years (18250 time-steps). Red lines represent activated unrecoverable damage, blue lines represent recoverable damage and black lines total damage (recoverable + unrecoverable). Some spikes on the recoverable damage curves are present. However such damage is usually recovered in healthy patients, as at the end of simulations the total damage is mostly 0.

**Figure 5:**
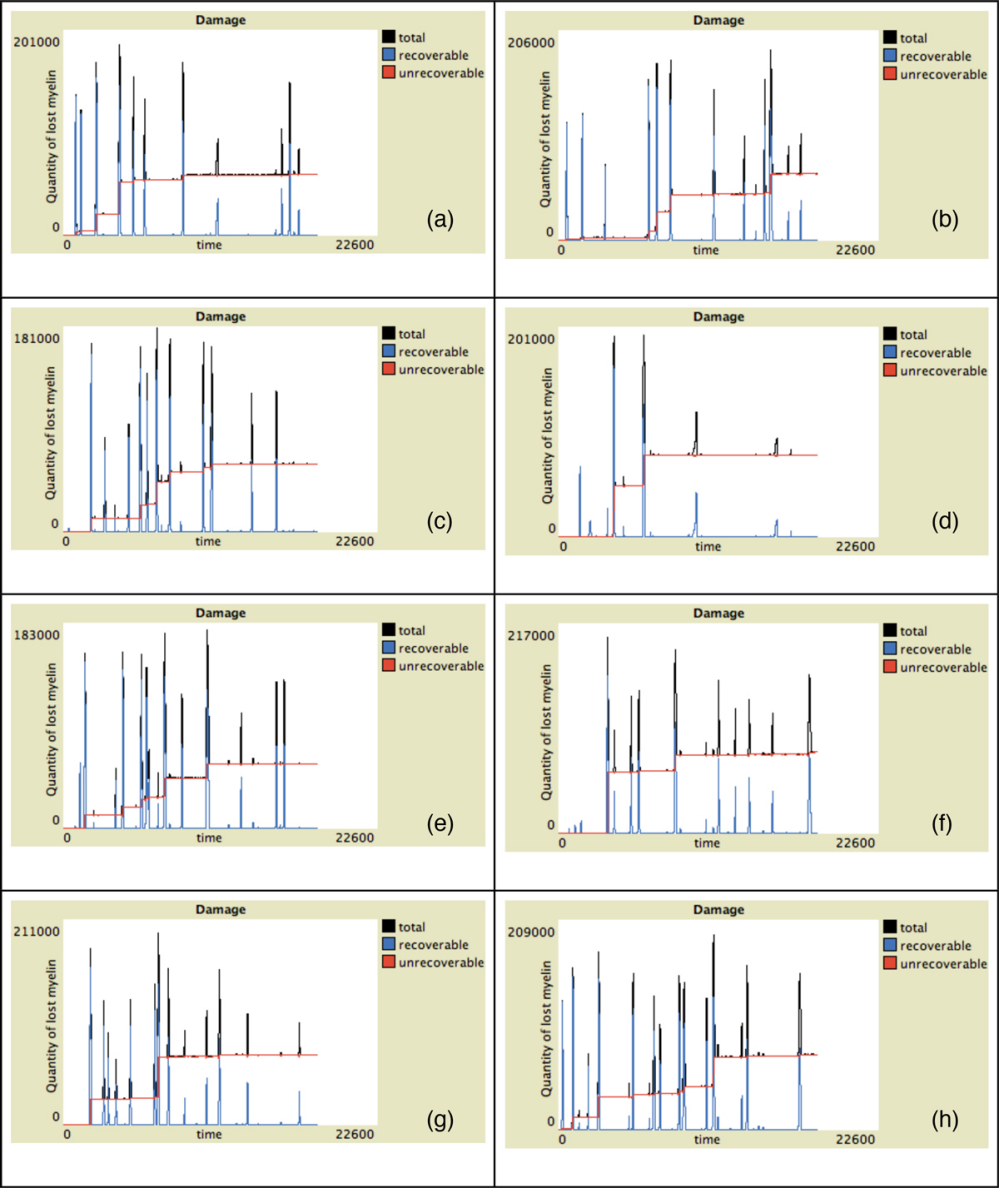
**Damage progression vs time in hill patients**. Simulation of eight randomly-selected hill virtual patients. Simulation time is 5 years (18250 time-steps). Red lines represent activated unrecoverable damage, blue lines represent recoverable damage and black lines total damage (recoverable + unrecoverable). In this case it is possible to observe more frequent spikes in the reversible damage curves. Furthermore unrecoverable damage (that can be correlated with the appearing MS plaques) is also present.

**Figure 6:**
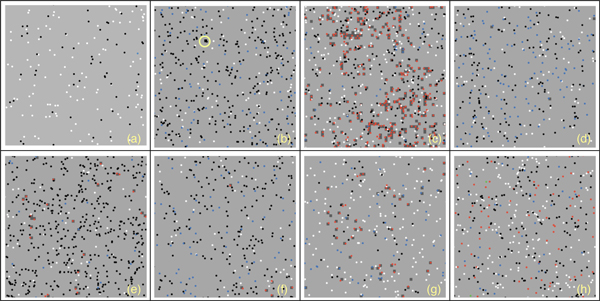
**Spatial plot at the end of the simulation in healthy patients**. Figure gives a spatial representation of the simulated scenario (i.e. a small portion of brain tissues) at the end of the simulation for eight randomly-selected healthy virtual patients. Light green patches represent non-damaged areas. Dark green patches (see for example plot (c)) represent areas with recoverable damage. Black patches (see for example plot (b), yellow circle) represent areas with unrecoverable damage. Red dots represent activated Teff and blue dots represent activated Treg. Green dots represent viruses. White and black dots represent resting Teff and Treg, respectively.

**Figure 7:**
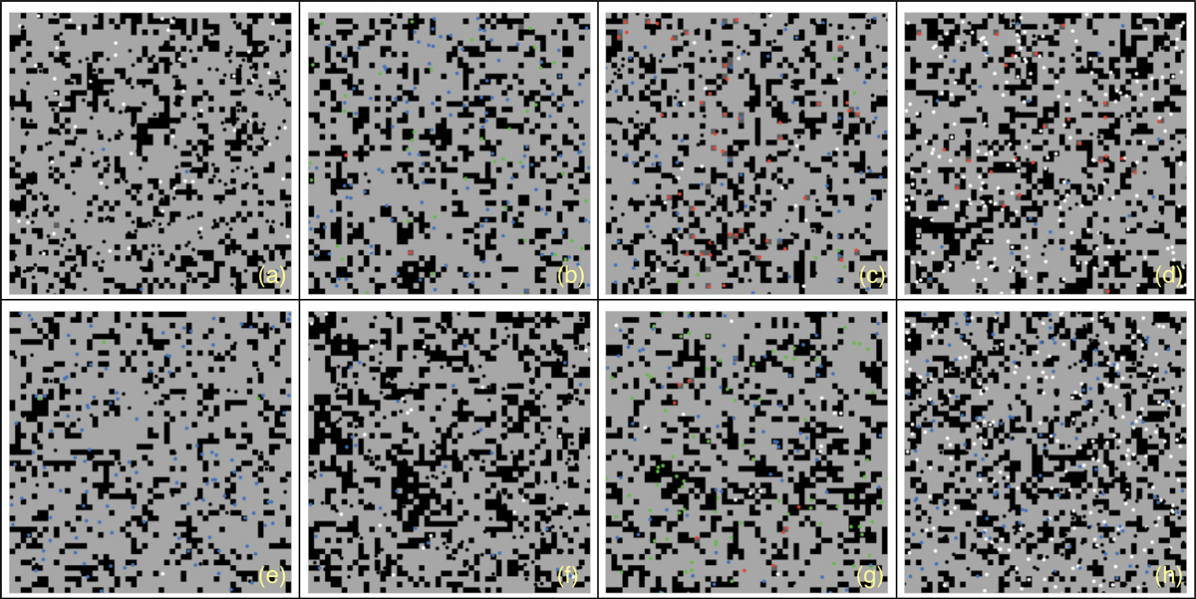
**Spatial plot at the end of the simulation in hill patients**. Figure gives a spatial representation of the simulated scenario (i.e. a small portion of brain tissues) at the end of the simulation for eight randomly-selected healthy virtual patients. Light green patches represent non-damaged areas. Dark green patches represent areas with recoverable damage. Black patches represent areas with unrecoverable damage. Red dots represent activated Teff and blue dots represent activated Treg. Green dots represent viruses. White and black dots represent resting Teff and Treg, respectively.

We also observed that (data not shown), in some cases (for some seeds) a decrease in the Teff or an increase Treg proliferation does not always entitle less severe relapses and, as a matter of fact, it could produce more severe relapses. This is mainly due to the stochasticity of the model. It may happen that the stochastic injection of new resting Teff may be not shortly followed by an equivalent injection of Treg, thus creating a temporary disequilibrium between the two populations which would entitle some neural damage even in potential healthy patients.

Results presented here suggest that presence of a genetic predisposition is not always a sufficient condition for developing the disease. Other conditions such as a breakdown of the mechanisms that regulate and allow peripheral tolerance should be involved. This has also been observed in [13]. In our case we supposed that a malfunctioning of self-reactive regulatory T cells caused by lower duplication rates was the cause. Of course other conditions may be the cause of such a malfunctioning.

Moreover we observed that in the simulations of hill patients relapses mainly occur in the first half of the simulation rather than in the second half (see, for example, Figure 5, plots (a),(c),(d) and (e)). This could be in line with clinical observations which showed that the relapse rate tends to decrease as the disease progression [14,15].

## Conclusions

In this work, we presented the first simple model of MS using agent based modeling approach. It shows the main behavior of this neural disease i.e., the relapse-remitting one. Compared to the real biological scenario, the model is naive, but takes into account the main entities involved at cellular level in the pathogenesis of MS. It allows to grasp how the role of regulatory vs effectors cells and their internal dynamics are crucial in the understanding the evolution of neural damage in the progression of the pathology. We will continue working with this model to integrate the damage done by demyelination and role of Treg cells in a quantitative way to correlate the disfunction of Treg cells and demyelinated area as shown in a recent study [57].

MS is a disease that involves practically all the immune system machinery, both at cellular, molecular and sub-molecular level. A detailed model would require a specialized framework and for this reason would be computationally expensive. The current version of Netlogo is probably not suitable to this purpose. We are working in this direction using a general framework for the immune system developed by us for other pathologies. Progress in this direction will be published in due course.

## Competing interests

The authors declare that they have no competing interests.

## Authors' contributions

MP: designed the model, analyzed data, developed the Netlogo model, performed numerical simulations, wrote the manuscript. AMR: designed the model, analyzed data, developed the Netlogo Model, gave biological knowledge, wrote the manuscript. LT: gave useful insights and wrote the manuscript. FP: conceived the application of an agent based simulator to MS, supervised the whole project and drafted the manuscript.

## Declarations

This work has been sponsored by Merck KGaA, a chemical and pharmaceutical company.

This article has been published as part of *BMC Bioinformatics *Volume 14 Supplement 16, 2013: Twelfth International Conference on Bioinformatics (InCoB2013): Bioinformatics. The full contents of the supplement are available online at http://www.biomedcentral.com/bmcbioinformatics/supplements/14/S16.
